# *In vitro* models for non-alcoholic fatty liver disease: Emerging platforms and their applications

**DOI:** 10.1016/j.isci.2021.103549

**Published:** 2021-12-04

**Authors:** Maria Jimenez Ramos, Lucia Bandiera, Filippo Menolascina, Jonathan Andrew Fallowfield

**Affiliations:** 1 Centre for Inflammation Research, The University of Edinburgh, The Queen's Medical Research Institute, Edinburgh EH16 4TJ, UK; 2 Institute for Bioengineering, The University of Edinburgh, Edinburgh EH9 3BF, UK; 3 Synthsys - Centre for Synthetic and Systems Biology, The University of Edinburgh, Edinburgh EH9 3BF, UK

**Keywords:** Cellular physiology, Bioengineering, Cell biology

## Abstract

Non-alcoholic fatty liver disease (NAFLD) represents a global healthcare challenge, affecting 1 in 4 adults, and death rates are predicted to rise inexorably. The progressive form of NAFLD, non-alcoholic steatohepatitis (NASH), can lead to fibrosis, cirrhosis, and hepatocellular carcinoma. However, no medical treatments are licensed for NAFLD-NASH. Identifying efficacious therapies has been hindered by the complexity of disease pathogenesis, a paucity of predictive preclinical models and inadequate validation of pharmacological targets in humans. The development of clinically relevant *in vitro* models of the disease will pave the way to overcome these challenges. Currently, the combined application of emerging technologies (e.g., organ-on-a-chip/microphysiological systems) and control engineering approaches promises to unravel NAFLD biology and deliver tractable treatment candidates. In this review, we will describe advances in preclinical models for NAFLD-NASH, the recent introduction of novel technologies in this space, and their importance for drug discovery endeavors in the future.

## Graphical abstract

**Figure:**
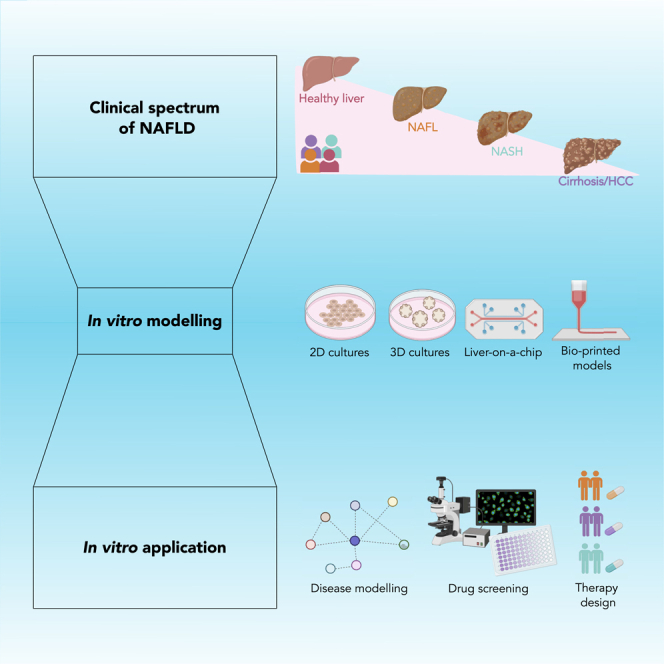

## Introduction

Non-alcoholic fatty liver disease (NAFLD) is the most common chronic liver disease, mainly affecting patients with obesity and type 2 diabetes mellitus (T2DM). NAFLD is the umbrella-term used for a range of conditions caused by a build-up of fat in the liver and encompasses isolated fatty liver (steatosis) and non-alcoholic steatohepatitis (NASH) with increasing degrees of liver inflammation, fibrosis (scarring), and potentially cirrhosis (end-stage fibrosis with vascular changes and functional impairment). Its worldwide prevalence is currently ∼25%, with 41% of the patients ultimately developing fibrosis (Younossi et al., 2016) and is projected to increase further by up to 18.3% in some developed countries by 2030 (Estes et al., 2018). Sedentary lifestyles and booming economic growth in the most developed countries sustain such trends, which see NASH becoming the leading cause of liver transplant (Zou et al., 2020).

To date, there are no licensed pharmacological treatments for NASH. While bariatric surgery is effective in a small proportion of patients (Lee et al., 2019), diet and exercise remain the cornerstone of disease management, due to evidence that a 10% body weight loss allows a reversion of steatosis, inflammation, and even fibrosis (Vilar-Gomez et al., 2015). However, the difficulty of achieving and sustaining this supports the need for disease-specific pharmacological treatment. Current medical treatment also comprises drugs for comorbidities related to NAFLD. Patients with or without T2DM may be treated with anti-diabetic medications. A recent systematic review of 18 trials (Blazina and Selph, 2019) presented evidence that supports the efficacy of some diabetes drugs in reducing liver fat and resolving NASH (e.g., pioglitazone, liraglutide), although associated weight gain with pioglitazone warrants caution. Blazina and Selph (2019) concluded that larger trials are needed to elucidate the benefits and risks of diabetes pharmacotherapy in NAFLD patients.

Given the complexity of NAFLD pathophysiology and the phenotypic heterogeneity, both genetic and environmental factors are thought to contribute to disease progression and susceptibility (Eslam et al., 2018). Several genes that participate in lipid droplet remodeling, hepatic very low density (VLDL) secretion and generation of predominant molecular species of phosphatidylinositol (PI) in cell membranes have been identified as NAFLD genetic variants. Specifically, the most well-described variants are patatin-like phospholipase domain-containing protein 3 (PNPLA3) I148M (Romeo et al., 2008), transmembrane 6 superfamily member 2 (TM6SF2) E167K (Kozlitina et al., 2014) and obesity-linked suppression of membrane-bound O-acyltransferase 7 (MBOAT7) (Mancina et al., 2016). The PNPLA3^*I*148*M*^ variant has been linked to NASH progression and fibrosis (Valenti et al., 2010; Rotman et al., 2010; Sookoian et al., 2009) and replicated in several independent genome-wide association studies.

Increasingly, it is suggested that a ‘one size fits all’ approach to treat NAFLD might not be optimal given the heterogeneity of the disease. Moreover, early pathways that participate in NAFLD progression are not commonly investigated in clinical trials, as the predominant focus is on NASH with moderate-to-severe fibrosis or cirrhosis. A comprehensive understanding of the whole disease spectrum –and not just the later stages– could be the key to understanding this disease and its progression.

Ahead of clinical trials, *in vivo* models remain a crucial tool to study NAFLD. Larger animal models such as rabbits (Ogawa et al., 2010), monkeys (Hansen et al., 2017) or minipigs (Pedersen et al., 2020; Lee et al., 2009) may have greater proximity to humans but can present ethical issues, are more difficult to handle and can be time-consuming and costly. While relying mostly on rodents, *in vivo* modeling has shown improved human translatability in studies of NAFLD pathogenesis and evaluation of potential therapeutic targets (Hansen et al., 2020; Clapper et al., 2013). However, rodent models also have disadvantages. Indeed, the human relevance and predictivity of laboratory mice is limited by species-specific biology and genetics (Reimer et al., 2020), coupled with physiological factors that result from growth in the laboratory, as opposed to a natural environment. Critically, this can affect the microbiome of mice and their potential response to drug therapies that are subsequently funneled through to human trials (Rosshart et al., 2019). Of note, the microbiome has been strongly linked to NAFLD pathogenesis (described in more detail in the following section).

*In vitro* models are, in many settings, a suitable alternative for studying NAFLD, as a result of increasing sophistication and the ability to recapitulate several hallmarks of the disease. A multi-disciplinary approach to the development of *in vitro* models led to solutions far more complex than the traditional two-dimensional (2D) cultures (e.g., liver-on-a-chip platforms and three-dimensional (3D) models). While supporting a better understanding of the disease, these models could be used as reliable drug screening platforms. In this review, we will detail which *in vitro* models have been used to date for NAFLD and NASH, discussing their advantages and disadvantages. We offer our perspective on future developments and translational opportunities in this field, which will be of relevance to researchers interested in the pathophysiology of NAFLD, drug development, and bioengineering.

## NAFLD pathogenesis

A detailed discussion of NAFLD pathogenesis is beyond the scope of this article; the interested reader is directed to recent comprehensive reviews (Loomba et al., 2021; Cariou et al., 2021). The current model of NAFLD-NASH pathogenesis is one of ‘substrate overload’, whereby the liver's capacity to handle the primary metabolic energy substrates (e.g., carbohydrates and fatty acids) is overwhelmed, leading to the accumulation of toxic lipid species.

Initially, the expansion of the subcutaneous adipose tissue in obese NAFLD patients leads to the accumulation of free fatty acids (FFA) in the muscles. This induces insulin resistance (IR), inhibiting glucose uptake (Brunt et al., 2015; Godoy-Matos et al., 2020). Concurrently, IR also compromises fat storage in adipocytes, causing FFA to be released into circulation and reaching the liver. Here, FFA induces hepatic IR, gluconeogenesis, very low-density lipoprotein (VLDL) release, as well as an increase of pro-inflammatory adipokines. To balance the elevated blood glucose levels, insulin, and FFA, the liver increases the storage of fatty acids and their synthesis through *de novo* lipogenesis, regulated by the sterol regulatory element binding protein-1 (SREBP1c) (Donnelly et al., 2005). Some sphingolipids, besides being correlated with IR, are also linked to hepatic oxidative stress and inflammation, suggesting their importance in NASH progression (Apostolopoulou et al., 2018).

The major difference between NAFLD and NASH is the presence of hepatocellular injury. In NASH, there is an increase of reactive oxygen species (ROS) and mitochondrial uncoupling, as a consequence of the metabolism of the fatty acids present in hepatocytes (Brunt et al., 2015). Hepatocyte ballooning (a special form of liver cell degeneration) has also been linked to the progression of NAFLD. These large cells with irregular cytoplasm show diminished expression of caspase-9 (conferring resistance to apoptosis) and enhanced production of Sonic hedgehog, a ligand of the hedgehog signaling pathway which promotes hepatic fibrogenesis (Hirsova and Gores, 2015). Kupffer cells (KC) and infiltrating macrophages contribute to hepatic inflammation, caused by the accumulation of fatty acids or damage-associated molecular patterns (DAMPs) released by the dying hepatocytes. They can activate toll-like receptors (TLRs) and the inflammasome, maintaining the inflammatory environment; and promote fibrosis through the activation of hepatic stellate cells (HSC). These cells play a critical role in fibrosis, and perpetuate inflammatory activity in the liver by increasing ROS and CCR5 levels, leading to extracellular matrix (ECM) deposition through stimulation by transforming growth factor β (TGFβ) (Pafili and Roden, 2020).

Gut microbiota dysbiosis (an “imbalance” in the gut microbial community) is also believed to play an important role in disease progression, as suggested by the distinctive microbiome signatures of NAFLD patients. Bacteria and their products, which reach the circulation and liver as the tight junctions of the intestinal barrier weaken, can trigger tissue and systemic inflammation. Some of these bacteria can release ethanol-like products, increasing ROS levels and inflammation, and hence promote NASH progression (Chen and Vitetta, 2020).

Although disease progression is variable in NAFLD, ∼5% will progress to cirrhosis, with an increased risk of hepatic decompensation, hepatocellular carcinoma (HCC), and liver-related mortality (Lindenmeyer and McCullough, 2018). Interestingly, HCC in NAFLD patients may also arise in non-cirrhotic liver (Huang et al., 2021). In a recent meta-analysis, non-cirrhotic NASH subjects were at greater risk of developing HCC than non-cirrhotic patients of other etiologies of liver disease (odds ratio 2.61) (Stine et al., 2018).

## *In vitro* culture systems in NAFLD

### Cell lines

Cell lines are widely used in research and drug development. When compared to *in vivo* models, cell lines can be easily cultured at large scale in a cost-efficient manner. Furthermore, they can be maintained longer than current NAFLD *ex vivo* models, which have a usual life-span of ∼5–15 days (Wu et al., 2018; Palma et al., 2019).

According to their origin, cell lines are either tumor-derived (commonly known as immortalized), primary cell lines, or pluripotent stem cells (PSC). Immortalized cell lines used for NAFLD research (e.g., HepaRG, THP-1 or LX-2), offering unlimited growth and stable phenotype, streamline standardized culture protocols and assay reproducibility (Ramboer et al., 2014). Nonetheless, mutations in immortalized cell lines can hamper translation of observations to the human *in vivo* condition; for instance, HepG2 is characterized by the PNPLA3 I148M mutation, which is known to affect some metabolic functions (Gunn et al., 2017). Primary human cells, isolated from liver tissue as hepatocytes or non-parenchymal cells (NPC), better resemble the *in vivo* phenotype (Wilkening et al., 2003). Indeed, harvesting of these cells from NAFLD patients enhanced the accuracy of drug metabolism studies (Ganji et al., 2015; Schwartz et al., 2020). However, their limited culture time and availability, especially for non-pathological (“healthy”) controls, as well as sample heterogeneity, are disadvantages (Zeilinger et al., 2016; Bell et al., 2018). When focusing on NAFLD modeling, inherent donor liver variability represents a double-edge sword, setting the basis for the investigation of genetic polymorphisms (Müller and Sturla, 2019). Lastly, PSC can be differentiated into liver-like cells from liver stem cells, induced pluripotent stem cells (iPSC) or embryonic pluripotent stem cells (ESC) (Hu and Li, 2015; Touboul et al., 2010; Takahashi et al., 2007). Cell differentiation, achieved via addition of growth factors and nutrients to the culture medium, results in phenotypic similarity to primary cell lines in addition to indefinite expansion in culture (Soret et al., 2021). Moreover, iPSC are the preferred PSC for investigation, the use of ESC being associated with ethical concerns (Zeilinger et al., 2016). Recent *in vitro* models with human PSC have been developed by Sinton et al. (2021); Holmgren et al. (2020); Coll et al. (2018) for NAFLD modeling. In Table 1, advantages and disadvantages of various cell lines used in NAFLD *in vitro* modeling are detailed.

**Table 1:** *In vitro* cell lines that have been used to investigate NAFLD, highlighting their advantages and disadvantages

Cell line	Description	Advantages	Disadvantages	References
HepG2	Human hepatocellular carcinoma cell line	– Cheap – Easy to maintain – Uptake and storage of exogenous FA – Uptake of lipoprotein remnants	– Low drug-metabolizing capacity – Rapid dedifferentiation – Transcript differences with human hepatocytes – Fetal phenotype – Cancer origin/phenotype – Low FA oxidation – PNPLA3 I148M mutation – Mainly LDL secretion	Green et al. (2015, 2020); Gunn et al. (2017); Jennen et al. (2010); Lőrincz et al. (2021); Srivastava and Chan (2008)
HepG2/C3A	Clonal derivative of HepG2	– Closer phenotype to human hepatocytes – Growth on glucose deficient medium – In liver spheroids, high sensitivity to drug-induced liver injury	– Strong contact inhibition of growth – Capable of growing on free-glucose medium – Cancer origin/phenotype – Lower metabolism than HepG2 – Low basal expression of CYP2E1	Baquerizo et al. (2015); Flynn and Ferguson (2008); Garcia et al. (2011); Gaskell et al. (2016)
HepaRG	Human hepatocellular carcinoma cell line	– Highly differentiation into hepatocytes – Expression of several CYP and phase II enzymes – Uptake and storage of exogenous FA – Secretion of lipoproteins – No PNPLA3 mutation	– Low levels of CYP2D6 and CYP2E1 – Cancer origin/phenotype – Overexpression of CYP3A4 – No bile collection	German and Madihally (2019); Green et al. (2020); Jennen et al. (2010); Lőrincz et al. (2021)
L02	Human fetal liver cell line	– Easy culture – Functional similarities with hepatocytes	– Fetal phenotype	Hu et al. (2013)
Huh7	Human hepatocellular carcinoma cell line	– Lipolytic enzyme expression – Effective CYP3A4 activity when confluent	– Fetal phenotype – Cancer origin/phenotype – Low FA oxidation on glucose – Steatosis overestimation	Green et al. (2020); Gunn et al. (2017)
Hepa1c1c7	Mouse hepatocellular carcinoma cell line	– High induction of CYP1A1 – Suitable for study of chemoprotective enzymes by organic selenium-containing compounds	– Cancer origin/phenotype	El-Sayed et al. (2007)
AML-12	Mouse liver cell line	– Contains human transgenic TGF-α – High expression of gap junction proteins	– Expression of liver-specific proteins decreases in long-term	Wu et al. (1994)
Hepa1-6	Mouse carcinoma cell line	– Suitable for drug evaluation – Favourable immune profile	– Fetal phenotype – Cancer origin/phenotype	Kachlishvili et al. (2020); Lacoste et al. (2017); Urs (2018)
RAW264.7	Mouse monocyte/macrophage-like cells	– Easy expansion in culture – High efficiency for DNA transfection – Sensitivity to RNA interference	– Genotypic and phenotypic drifts due to repeated passaging (until passage no. 30)	Hartley et al. (2008); Taciak et al. (2018)
THP-1	Human monocytic cell line	– High growth rate – High reproducibility	– Lack of surface and cytoplasmic immunoglobulin – Dependent on M-CSF or similar for differentiation into macrophages – Genotypic and phenotypic drifts due to repeated passaging (until passage no. 25) – Differences with human phenotype	Chanput et al. (2014)
Bone marrow-derived macrophages	Mouse primary macrophage cells	– Homogeneous distribution – High proliferative capacity – Transfectable	– Dependent on M-CSF or similar for differentiation into macrophages – Shorter life-span	Weischenfeldt and Porse (2008)
LX-2	Hepatic stellate cells (HSCs) from normal liver	– Activated phenotype with fibroblast-like appearance – Inducible retinoid metabolism – High transfectability – Growth in low-serum conditions	– Low-serum/basement matrix required for quiescent phenotype – Genotypic and phenotypic drifts due to repeated passaging (until passage no. 50)	Vande Bovenkamp et al. (2007); Weiskirchen et al. (2013); Xu et al. (2005)
T6 stellate cells	Rat primary HSCs from liver cell line	– Inducible retinoid metabolism – Morphologic and proliferative characteristics from activated HSCs – Form cytosolic lipid droplets	– Retinoid protein expression and processing similar to quiescent cells – Low-serum/basement matrix required for quiescent phenotype	Van de Bovenkamp et al. (2007); Vogel et al. (2000); Xu et al. (2005)
Primary cell lines	– Primary hepatocytes – Primary KC – Primary HSC	– Isolation from NAFLD/NASH patients is possible – Interindividual variation studies are possible – FA oxidation – High functionality – Human metabolism is reproduced	– Difficult isolation – Low availability – Lose differentiation in long-term – Lose proliferation in long-term – Difficult reproducibility	Brandon et al. (2003); Green et al. (2020); Wilkening et al. (2003)
3T3-L1 MBX	Mouse fibroblast	– Adipocyte differentiation – Robust response to insulin – Present lipid droplets	– Low proliferation rate	ATCC; Berger and Géloën (2019)
3T3-J2	Embryonic mouse fibroblast	– Expression of genes present in liver – Use in multicellular aggregates to enhance liver functions – Stabilisation of liver phenotype without NPC is possible	– Non-liver source – Fibroblast density modulates hepatic function in dose-dependent manner	Bhatia et al. (1998); Ware et al. (2021)
Liver sinusoidal endothelial cells (LSEC)	Primary human cell line	– High endocytosis capacity – High permeability – Long life-span	– Low availability – Difficult isolation – Low proliferation rate – Rapid loss of differentiation	German and Madihally (2019); Poisson et al. (2017)
Human umbilical vein endothelial cells (HUVEC)	Human non-pathological tissue	– Generation of vascular-like endothelial network when cultured with hepatocytes in spheroids – Easy access – Established protocols for tissue culture in a serum-free medium	– Non-liver source – Less migration of lymphocytes than LSEC – Reduced viability when co-cultured with HepaRG than LSEC	German and Madihally (2019); Medina-Leyte et al. (2020)
Caco-2	Human epithelial cells from colorectal adenocarcinoma	– Spontaneous change in enterocytic characteristics at confluence – Tight junctions between cells	– Genotypic and phenotypic drifts due to repeated passaging – Presence of multilayer zones	Briske-Anderson et al. (1997); Poncede León-Rodríguez et al. (2019)
NCTC-1469	Mouse NPC from liver cell line	– Expression of estrogen receptors		Asahi et al. (2010)
Pluripotent stem cells (PSC)	Stem cells that can differentiate into endoderm, mesoderm or ectoderm	– Differentiation into desired cell type – Similar phenotype to primary cell lines – Unlimited culture time	– Fetal phenotype – No standardized differentiation protocol – Epigenetic memory may prevent differentiation – Differentiation is time consuming – Cost	Soret et al. (2021); Zeilinger et al. (2016)

### Cell culture models

**Figure 1:**
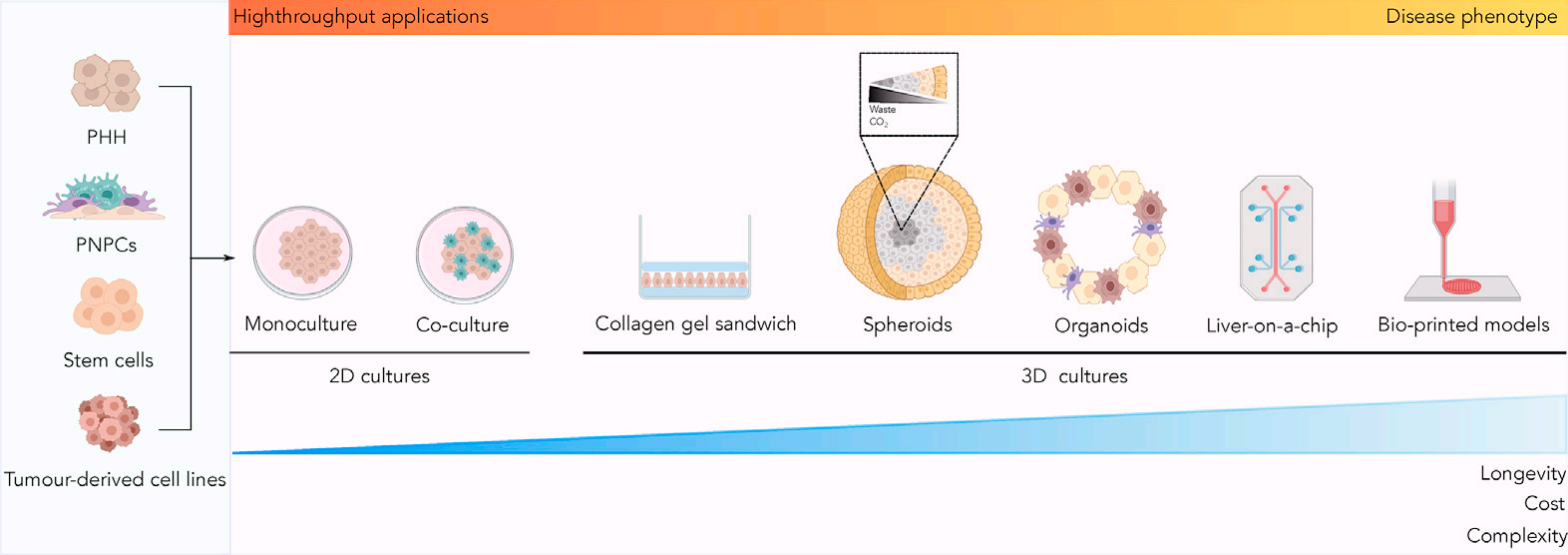
Cell sources and *in vitro* models used in NAFLD studies Primary human hepatocytes (PHH), primary non-parenchymal cells (PNPC), stem cells and tumor-derived cell lines (cyan panel) can all be used to create two-dimensional (2D) and three-dimensional (3D) models for NAFLD (pink panel). 2D cultures (e.g., monocultures and co-culture) are denoted by a single or several cell types growing in a monolayer. 3D cultures (e.g., collagen gel sandwich, spheroids, organoids or liver-on-a-chip) have been recently introduced to elucidate NAFLD, and their development can rely on bio-printing technology. Self-aggregated spheroids present an accumulation of carbon dioxide (*CO*_2_) and waste in their core. Cell culture models are listed by increasing cost, longevity and complexity. While complexity correlates with ability of the model to accurately capture the disease phenotype, simpler models are better suited to high-throughput applications.

2D and 3D cultures have been extensively used in NAFLD research and *in vitro* modeling (Figure 1, Table S1), albeit to a different extent. We have performed a systematic review to observe the evolution and focus of *in vitro* models in NAFLD (Figure S1). Our systematic review indicated that researchers predominantly favored 2D monocultures (59.4%) to more complex models (2D co-cultures (14%), spheroids (9.7%), organoids (7.3%), liver-on-a-chip (7.8%), collagen gel sandwiches (1.2%), and micropatterned cultures (0.6%). When analyzing the selection of cell culture systems over time (Figure S2), we observed an increasing trend in the publication of 3D *in vitro* models, specifically in on-chip cultures. This suggests 3D culture systems are becoming more relevant in this field. Focusing on the large number of publications in monocultures in 2020, we note that these systems have been used in conjunction with additional *in vivo* experiments, or as a benchmark for more sophisticated *in vitro* models. As an example, the insert in Figure S2 distinguishes publications in 2020 that have used monocultures as the only *in vitro* experiment from those that used them in combination with other cell culture models. We speculate that technological advances, standardizing the development of more complex and affordable solutions, might underlie this observation.

As reported above, 2D monoculture is the most established NAFLD *in vitro* model. In this system, steatosis induction is routinely performed by adding a mixture of unsaturated and saturated FFA to the culture medium. Typically, oleic acid (OA) and palmitic acid (PA) are selected, being the most abundant in humans (Hodson et al., 2008). According to our systematic search the most commonly used ratio for these FFA is 1:2 (PA:OA). This FFA ratio exposure emulates dietary conditions that are commonly observed *in vivo*, inducing hallmarks of NAFLD-NASH (e.g., cytoplasmic accumulation of TG in hepatocytes, ER stress, inflammation, and cell death) (Gomez-Lechon et al., 2007).

The low cost, ease of use, and suitability for high-throughput screening make 2D monocultures attractive for disease modeling. Here, the choice of cell sources constrains the NAFLD mechanistic studies enabled by the *in vitro* model. For example, Lyall et al. (2018) showed that hepatocyte-like cells derived from human ESC retained the ability to form the cytosine modification 5-hydroxy-methylcytosine (5hmC), rapidly lost when culturing HepG2, providing a means to elucidate the role of epigenetic dysregulation in NAFLD pathogenesis. Similarly, an hPSC-derived model was used to study the re-wiring of mitochondrial metabolism in early steatosis (Sinton et al., 2021). Monoculture models for NAFLD have also been valuable for investigating metabolic and fibrotic pathways (Graffmann et al., 2016; Kanda et al., 2018; Zhang et al., 2016), evaluating therapeutic compounds (Boeckmans et al., 2020; Armstrong et al., 2016; Anfuso et al., 2020), and assessing hepatic drug metabolism/toxicity (Gomez-Lechon et al., 2003).

Notwithstanding the advantages outlined above, 2D monocultures are unable to faithfully mimic *in vivo* NAFLD conditions due to the lack of interaction with NPC and disturbances in the extracellular environment. An improvement over this system is provided by 2D co-cultures, typically involving two cell types, such as hepatocytes and NPC, growing in a monolayer. As with monocultures, the steatotic phenotype can be induced upon treatment with FFA. Interestingly, a co-culture of Huh7 and LX-2 enabled Barbero-Becerra et al. (2015) to demonstrate the key role of hepatocyte-HSC crosstalk in the activation of HSC, a key feature of NASH. Co-cultures including macrophages (Zhong et al., 2017; Chen and Ma, 2019) have also elucidated the role of multiple inflammatory pathways in NAFLD and NASH.

NAFLD and NASH can be induced in 2D co-cultures of PHH and iPSC (Parafati et al., 2018), yet their capacity as accurate *in vitro* models decays with time as they lose their xenobiotic metabolism and hormone response (Gurevich et al., 2020). 3D cultures can enhance these features, allow cell-cell interactions and cell polarity (Treyer and Müsch, 2013; Godoy et al., 2013). They can provide new insights into complex physiological mechanisms and potential therapeutic targets, increasing our knowledge of NAFLD. Furthermore, they represent a suitable alternative to *in vivo* models at early stages of drug development (Romualdo et al., 2021; Beisner et al., 2021; Eilenberger et al., 2020). The main 3D *in vitro* models used for NAFLD –spheroids, organoids, and microfluidic devices– are detailed in the following section.

We classified the NAFLD *in vitro* models identified in our systematic search, according to their intended application, into NAFLD biology studies and compound testing. 47.9% of the publications relied on *in vitro* models to elucidate the role of different proteins, pathways or hallmarks of the disease; 18.8% of the papers detailed the development of new *in vitro* models to better mimic NAFLD biology and its progression; while 33.3% of the studies assessed the therapeutic potential of putative compounds and drug targets for NAFLD and NASH.

**Figure 2:**
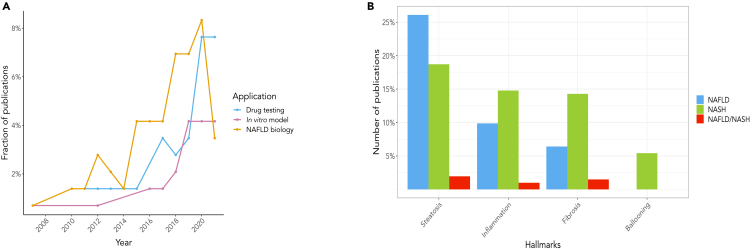
Systematic analysis of the scientific literature on NAFLD *in vitro* cell culture models published between 2007 and 2021 (A) Trend over time of scientific publications centered on NAFLD *in vitro* models, clustered by application. Despite the majority published studies using *in vitro* models focused on NAFLD biology (orange), the past years have seen a marked increase in scientific articles presenting novel *in vitro* models (purple) or using available culture systems for compound testing (cyan). The concurrent decrease in investigations of the role of specific pathways and/or proteins in NAFLD pathogenesis suggests a shift in focus and efforts within the scientific community. (B) Barplot showing the percentage of *in vitro* models that have been used to study steatosis, inflammation, fibrosis, or hepatocellular ballooning. For each disease hallmark, publications are clustered, according to their focus, in NAFLD (cyan), NASH (blue) or both NAFLD and NASH (dark blue). Steatosis is the most studied feature in NAFLD and NASH, followed by inflammation and fibrosis. Hepatocellular ballooning is the least studied feature in the scientific literature. For more information regarding the historical perspective of the evolution of *in vitro* models for NAFLD. See also Figure S2.

Despite a larger number of studies focused on the biology of NAFLD, the last 3 years have seen an increase in publications reporting compound testing (Figure 2A). This, together with the simultaneous decline in the study of disease mechanisms in NAFLD, suggests a possible shift in research on the condition. Specifically, recent publications leverage available knowledge on NAFLD pathogenesis to devise strategies for treatment. We explored whether this shift in the field was promoted by a greater involvement of pharma, but did not observe any direct link, as most of the investigations remain hosted in academic settings. We also noted that despite the divergence in their severity and risk of progression, comparable effort has been devoted to NAFLD (51.4% of the studies) and NASH (43.7%). Only 4.9% of the investigations proposed *in vitro* models to mimic both conditions. Considering the varied clinical outcomes within the disease spectrum, it would be preferable to consider both NAFLD and NASH when investigating the disease. This is of paramount importance for compound testing since the mechanism of action (and potential efficacy) of drugs will depend on the disease stage.

In Figure 2B the distribution of the hallmarks of NAFLD/NASH across the studies is reported. Most publications concentrated on steatosis, followed by inflammation and fibrosis. The focus on steatosis is reflected by the predominant use of monocultures of hepatocytes. Interestingly, hepatocellular ballooning has barely been explored in these studies. Although it is difficult to objectively measure the presence of ballooned hepatocytes in patients' biopsies (Kakisaka et al., 2018), they are a pathognomonic histological feature of NASH that should be considered when studying this disease.

## 3D tissue culture models in NAFLD

Three-dimensional cultures are emerging as a bridge between the easy-to-use 2D cell cultures and more complex *in vivo* models. By maintaining hepatic cell types within a controlled microenvironment, 3D liver models better mimic the *in vivo* organ phenotype. From spheroids composed of a single cell type to multicellular aggregates including NPC and immune cells, 3D cultures can model the liver organotypic structure, retaining paracrine cell-cell interactions and metabolic functions for several weeks. Moreover, these models can be used to study fluid flow and perfusion when cultured on a chip. In this section, we will review the 3D culture models most recently proposed to recapitulate NAFLD. Specifically, we will detail the latest developments in spheroids, organoids, liver-on-a-chip and bio-printed platforms.

The collagen gel sandwich has been extensively used to culture liver cells (Engl et al., 2004; Sandwich, 2006; Kono et al., 1997; Choi and Choi, 2013; Wang et al., 2004). In this model, hepatocytes are seeded between two layers of collagen gel. The obtained structure better resembles the *in vivo* conditions, as demonstrated by reorganization of the cytoskeleton, improved morphology and polarity, and enhanced expression of liver-specific functions (Berthiaume et al., 1996). Within this model, hepatocytes maintained secretion of hepatic compounds for at least six weeks; a three-fold increase in the secretion window offered by monolayer collagen gel cultures (Dunn et al., 1991). Another collagen gel sandwich model, combining hepatocytes with liver sinusoidal endothelial cells (LSEC), mimicked the liver lobular architecture and showed stable secretion and metabolic activity for up to 4 weeks (Bale et al., 2015). While allowing long-term monitoring, the collagen gel sandwich has limitations. Indeed, when compared to 3D spheroids, a PHH-based collagen gel sandwich does not prevent hepatocyte dedifferentiation, as evidenced by perturbed glycolysis and gluconeogenesis, and shows lower sensitivity to long-term exposure to hepatotoxic compounds in cytotoxicity studies (Bell et al., 2018). Moreover, the collagen thickness may hinder cell-cell interactions (Soret et al., 2021). For these reasons, this model is seldom used to investigate NAFLD.

**Figure 3:**
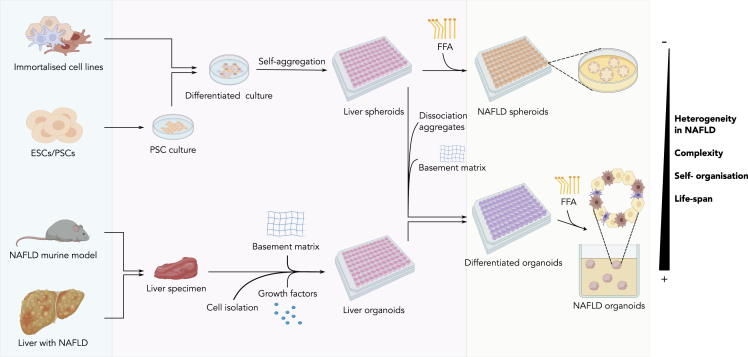
Development protocol for spheroids and organoids Both models can be obtained from different sources (cyan panel) -immortalized cell lines, embryonic stem cells (ESC), pluripotent stem cells (PSC), NAFLD murine models or liver from NAFLD patients. Immortalized cell lines and differentiated ESC/PSC self-aggregate to form spheroids. To induce the NAFLD phenotype, free fatty acids (FFA) supplementation is required. To form liver organoids, cells from liver specimens are extracted and isolated. Hence, they are exposed to selected growth factors in presence of a basement matrix. These liver organoids then differentiate into the specific cell lines and acquire the NAFLD phenotype upon FFA supplementation (light green panel). In addition, differentiated organoids can also be obtained from ESC/PSC or immortalized cell lines aggregated in liver spheroids. After dissociation of the aggregates and subsequent cultivation with a basement matrix, the differentiated organoids are ready to be used for NAFLD studies. The heterogeneity of the disease, its complexity, the self-organization of the cells or the life-span of the cultures is higher in organoids than in spheroids.

More recently, spheroids and organoids have become the 3D *in vitro* models of choice for studying NAFLD (Soret et al., 2021). Spheroids and organoids differ based on the origin and number of cell types in the aggregate, the culture environment or the complexity of their structure (Kang et al., 2021). In this review, we refer to spheroids as 3D structures produced by free-floating, spontaneous self-aggregation of cell lines in the presence or absence of an ECM that can recapitulate some functional aspects of the liver. In contrast, we denote organoids as 3D systems obtained by embedding tissue stem cells, progenitor cells or tissue-resident cells isolated from liver specimens in a scaffold that imposes a liver-like spatial organization, thereby enabling modeling of the organotypic structure and function (Kang et al., 2021; Gunti et al., 2021). Figure 3 details how both NAFLD spheroids and organoids can be obtained from different sources.

### Spheroid models

Since the first spheroid model in the 1970s (Sutherland et al., 1971), multiple methods have been developed to establish systems of increasing sophistication. In NAFLD modeling, the most popular method for spheroid culture is liquid overlay, where cell suspensions are cultured on ultra low-adhesive surface plates and formation of spheroids can be monitored in real-time (Gunti et al., 2021). Alternative methods for spheroid assembly have been extensively reviewed in the scientific literature (Kang et al., 2021; Gilazieva et al., 2020; Ryu et al., 2019).

The simplest hepatic spheroid model consists of a single cell type, usually HepG2 or PHH. It is worth noting that spheroids support culturing of differentiated PHH for a minimum of 3–4 weeks. Upon treatment with pathological concentrations of FFA, carbohydrates, cyclosporin A or insulin, hepatic spheroids enabled induction and investigation of steatosis for up to 5 weeks (Damelin et al., 2007; Bell et al., 2016; Kozyra et al., 2018). Specifically, these models have helped to elucidate the role of hepatocytes in NAFLD (Damelin et al., 2007; Chong et al., 2020), the mechanism by which IR elicits *de novo* lipogenesis and lipid accumulation (Kozyra et al., 2018), the identity of putative molecular targets or the pharmacological action of novel compounds to ameliorate the disease (Chong et al., 2020; Eilenberger et al., 2020; Bell et al., 2016). Furthermore, the use of PHH in spheroids (Bell et al., 2016; Eilenberger et al., 2020) has shed light on the relevance of inter-individual variations as a determinant of susceptibility to steatosis development and progression (Dongiovanni et al., 2015). Thus, PHH-based spheroids could provide an ideal platform to demonstrate the impact of genetics on the variability in drug responses (efficacy and toxicity) among patients; the major cause of withdrawal of drugs from the market (Sim and Ingelman-Sundberg, 2011).

By co-culturing hepatocytes with NPC, beyond enhancing hepatocyte function *in vitro*, researchers can generate liver-like structures that recover the relevant mechanistic steps in NAFLD-NASH evolution (e.g. inflammation, HSC activation, and fibrogenesis). Hepatoma cell lines have been co-cultured with primary HSC to define pathways linked to liver steatosis and fibrosis (Salloum et al., 2021; Hurrell et al., 2020). Specifically, these co-culture systems showed enhanced collagen production, activation of HSC, upregulation of genes associated with fibrosis progression – such as collagen type I alpha I (COL1A1), TIMP metallopeptidase inhibitor 1 (TIMP1) or actin alpha 2 (ACTA2), and induction of pro-inflammatory cytokines. Bell et al. (2016) also co-cultured PHH-based spheroids with KC, HSC, and biliary cells, and highlighted that the co-culture systems enable modeling of an inflammatory response, as measured by an increase in IL-6 secretion. Another example of a multicellular spheroid model has been proposed by Kachlishvili et al. (2020). The researchers combined a hepatocyte cell line with macrophages to study the effect of low-dose trans-resveratrol on lipid metabolism and pro-inflammatory processes. They showed a decrease in lipid accumulation due to enhanced poly(ADP-ribose) polymerase (PARP) activity, following treatment with the compound. As previous studies in monocultures hinted at an environment and cell-type dependent effect of resveratrol on metabolic pathways (Lan et al., 2017), the study by Kachlishvili et al. (2020) demonstrated the relevance of 3D spheroids for investigating the mechanism of action of drug candidates.

Spheroid models have further enhanced our understanding of genetic variants linked to NAFLD. In Tanaka et al. (2021), HepG2-HSC spheroids were used to evaluate the effect of the MBOAT7 variant on inflammation in NAFLD. Silencing MB0AT7 expression with siRNA, the authors demonstrated a positive correlation between the presence of the variant and collagen expression, activation of HSC and expression of inflammatory and profibrotic cytokines. A similar system proved relevant to investigate the most common genetic variant in NAFLD patients, PNPLA3 I148M (Pingitore et al., 2019), and study the mechanisms involved in the early stages of fibrogenesis induced by lipid accumulation. The genetically predisposed spheroids were characterized by high fat accumulation and collagen production. This 3D model also captured antifibrotic effects following exposure to the compounds liraglutide and elafibranor. The same model was later selected as a screening platform for small molecule inhibitors, leading to the identification of momelotinib as a putative drug against the PNPLA3 I148M variant (Schwartz et al., 2020). PHH spheroids were additionally used to study the effect of the TM6SF2 E167K variant on steatosis establishment and progression *in vitro* (Prill et al., 2019). While the mutation led to higher intracellular lipids and upregulation of cholesterol biosynthesis and *de novo* lipogenesis, downregulation of genes implicated in NAFLD (e.g., ACADS, PNPLA2) revealed discrepancies with observed *in vivo* pathogenic mechanisms. However, the conclusions drawn from this study are limited by the small sample size exhibiting the TM6SF2 E167K variant (2 out of 5 PHH donors); a common challenge when working with PHH.

Despite their potential for disease modeling and drug efficacy testing, stem cell-based models are still mainly used in 2D cultures. Holmgren et al. (2020) assembled PSC-derived hepatocytes and HSC in spheroids, thereby avoiding the nuisance factor of spontaneous HSC activation induced by the stiffness of the ECM in 2D co-cultures (Wells, 2005). Gene expression analysis revealed similarities with PHH in glucose, lipid and cholesterol metabolism, reinforcing its suitability for NAFLD research. However, limited urea secretion and downregulation of enzymes involved in phase I and II drug metabolism hindered its use for drug screening studies.

Notwithstanding their simplicity and low cost, spheroid models offer limited control over their assembly and size when cultured in standard non-adhesive plates, presenting a scalability and reproducibility issue for liver spheres. Indeed, clusters of spheroids tend to merge into larger aggregates, challenging cell viability in culture by hampering both the intake of nutrients and oxygen diffusion (Underhill and Khetani, 2018a). To overcome this drawback, protocols detailing automated assembly of liver spheres have been published (Meseguer-Ripolles et al., 2021). In addition, novel approaches have incorporated the inclusion of hydrogel scaffolds, which direct the assembly of spatially isolated, controlled-size spheroids, in the development protocol (Ryu et al., 2019).

### Organoid models

Liver organoids are 3D culture models obtained by embedding tissue stem cells, progenitor cells or tissue-resident cells isolated from liver specimens within a hydrogel ECM. Their development protocol allows tighter control over the spatial organization of the multicellular aggregate, better approximating the heterogeneity and organotypic structure observed *in vivo* (Gunti et al., 2021); once the exposure to growth factors and support matrices enabling differentiation of multiple cell types has been defined. Organoids supersede the ability of 2D cultures to model (patho)physiological processes, spanning cell differentiation, migration and cell-cell interactions. Furthermore, they retain genomic stability and, owing to wide expansion in culture, are suitable for long-term storage and high-throughput screening of drugs (Soret et al., 2021).

Murine-derived organoids have been widely used to model the NAFLD phenotype. After feeding wild-type or genetically modified mice with high-fat containing or specialized diets (such as the methionine and choline-deficient diet), researchers can harvest liver tissue for cell isolation and culture the cells within a hydrogel ECM stimulated with selected stem cell media or growth factors (Broutier et al., 2016). Recently, Elbadawy et al. (2020) modeled NAFLD progression via murine organoids assembled with hepatocytes and activated HSC isolated from animals at three distinct disease stages (mild, moderate and severe NASH) (Elbadawy et al., 2020). In doing so, Elbadawy et al. established a model to investigate the effect of genetic stability and lipid metabolism on NAFLD progression. The authors noticed larger size and enhanced release of pro-inflammatory cytokines in early stage organoid models, compared to later stage ones. Other murine organoids have been used to decipher liver metabolism and its response to therapeutic compounds. For example, Pittol et al. (2020)explored the dependency of metabolic functions on Farnesoid X receptor (FXR) isoforms; Sano et al. (2021) elucidated the associations among free amino acids, FFA and the Keap1-Nrf2 system – involved in the principal protective response to oxidative and electrophilic stresses (Turpaev, 2013) – in lipotoxic hepatocytes, while Liu et al. (2021) investigated the use of uridine to ameliorate NAFLD. Despite notable successes, murine-based organoids can only approximate the pathophysiology of human NAFLD (Kim et al., 2020), suggesting a human organoid model might provide a more suitable research platform.

Several approaches for the development of human organoids to model NAFLD (and its extra-hepatic organ involvement) have recently been published. Ouchi et al. (2019) created a model from human PSC by co-differentiating epithelial and stromal lineages to form spheroids, later embedded in Matrigel and cultured with retinoic acid and maturation media. This protocol successfully yielded NPC including HSC-like cells, Kupffer-like cells and biliary stem cells; as well as hepatocyte-like and cholangiocyte-like cells, as confirmed by single-cell transcriptomics and FACS analysis. Upon FFA exposure, the organoids acquired a NAFLD phenotype denoted by steatosis, inflammation, hepatocyte ballooning and collagen production; hallmarks of NASH. Gurevich et al. (2020) opted instead for iPSC-derived cryopreservable hepatocytes sourced from NASH donors for organoid development. When co-cultured with physiologically-relevant ratios of isogenic HSC-like and Kupffer-like cells on collagen-coated ultra-low binding plates, the hepatocytes assembled in organoids amenable to observation for over 10 days. This system, characterized by the formation of bile canaliculi, proved useful for drug metabolism studies. In alignment with scientific evidence (Teo et al., 2014), efficient hepatocyte differentiation was promoted via glycogen synthase kinase 3 (GSK3) inhibition, which is known to induce endoderm differentiation. Similarly to Ouchi et al. (2019), treatment with FFA induced a dose-dependent accumulation of intracellular lipids and establishment of a NASH phenotype. McCarron et al. (2021) instead developed bipotent ductal organoids derived from end-stage NASH patient liver explants, with normal donor livers as controls. Strikingly, NASH patient-derived organoids were highly diverse in terms of metabolic and pro-inflammatory pathways and response to drugs. Moreover, the model was characterized by increased sensitivity to apoptosis, increased FFA induced lipid accumulation, and reduced albumin secretion– all typical features of advanced NASH. Another hepatic organoid model was developed using posterior foregut and hepatic endoderm. FFA induction elicited increasing lipid peroxidation and ROS. Interestingly, this 3D model highlighted disruption of the bile canalicular network with disease progression (Ramli et al., 2020).

Several organoid models recapitulating NAFLD phenotypes have used bulk RNA sequencing (RNA-seq) to identify gene signatures and pathways associated with the disease (McCarron et al., 2021; Elbadawy et al., 2020; Beisner et al., 2021; Ouchi et al., 2019). Yet, only a few have included flow cytometry (Ouchi et al., 2019; Gurevich et al., 2020; Ramli et al., 2020; Abbey et al., 2020) or single-cell RNA-seq (Ouchi et al., 2019) in their research to determine the proportion of the different cell types present in these organoids. Quantifying such ratio might inform protocols offering tighter control over the development of these 3D systems, and help address two open challenges: heterogeneity and low reproducibility (Garreta et al., 2020).

The organoid technology, especially when based on human stem cells, holds great promise for investigating NAFLD pathophysiology due to the ability to overcome confounding factors introduced by animal models. Thanks to enhanced stability and complexity, organoid models offer a viable platform for future drug development pipelines. The establishment of new cryopreservation (Gurevich et al., 2020) and reproducibility (Nuciforo and Heim, 2020) standards will certainly aid in unlocking the potential of organoids to offer a preclinical alternative to animal models in the near future. Indeed, validated organoids could provide an easy to image and more accurate model of human NAFLD. Recently, Takebe et al. (2017) targeted organoids reproducibility by establishing a technological platform to manufacture standardized liver buds starting from 3 committed progenitors on a large scale (>10^8^).

We note that the bioengineering field is boosting efforts toward controlling organoids' cellular complexity, providing vascular networks, and enhancing organoid maturation (Garreta et al., 2020). For example, Guye et al. (2016) showcased the use of heterologous GATA-binding factor 6 (GATA6) expression to trigger co-differentiation events within an iPSC population and obtain, within 2 weeks, a complex tissue with liver-like phenotype. In Velazquez et al. (2021), engineering of gene regulatory networks in PSC-derived human liver organoids enabled advancement of maturity and vascularization of the obtained *in vitro* system. In addition, gene editing using CRISPR/Cas9 has also been used in bioengineering to alter and study the function of specific genes. For example, Abbey et al. (2020) deleted Tribbles-1 (TRIB1) – a gene associated with NAFLD, in human iPSC which were then used to create hepatic spheroids.

### Liver-on-a-chip

Despite having proved informative for the study of NAFLD and complex diseases, a major drawback of the static 3D cultures is the lack of long-term control over fluid shear stress, nutrient/gas exchange or waste removal. To enable the automated control of such factors, microfluidic technology and liver-on-a-chip approaches have been adopted to model the liver microenvironment (Underhill and Khetani, 2018b). While the cost and complexity of microfluidic setups constitutes a barrier to their diffusion in laboratories, the standardized fabrication of chips favors reproducibility, a hurdle in organoid research.

Several liver-on-a-chip systems have recently been proposed to study NAFLD. The first appearing in the scientific literature approximated the liver architecture and microvasculature by culturing HepG2 cells in parallel microchannels mimicking the endothelial barrier (Gori et al., 2016). This microfluidic device demonstrated the ability of FFA perfusion to recapitulate steatosis. Since then, the combination of multiple cell types made on-chip cultures amenable to the study of cell-cell interactions, better reflecting the complexity of the hepatic microenvironment. Suurmond et al. (2019) co-cultured HUVEC with GelMa-encapsulated HepG2 to investigate NAFLD pathogenesis. Similarly to Gori et al., their system, distinguished by high cell viability and homogeneous cell distribution, enabled monitoring of steatosis establishment. Inclusion of KC in this *in vitro* model led to enhanced ROS expression and proinflammatory cytokine release. While HepG2 are a widely used cell line to investigate regulation of drug-metabolizing enzymes, PHH are the preferred choice in biotransformation studies, thanks to their *in vivo*-like functionality (Wilkening et al., 2003).

In light of their similarities to PHH, researchers increasingly based *in vitro* models on HepaRG cells. Suurmond et al. (2019) highlighted that by substituting HepG2 with HepaRG cells, their liver-on-a-chip displayed elevated levels of proinflammatory cytokines and improved reversibility of steatosis. HepaRG, in conjunction with KC, HUVEC and HSC encapsulated in GelMA, were used in the microfluidic model proposed by Cho et al. (2021). Through a 21-day life-span, the system recapitulated NASH features and showed neovascularization. Freag et al. (2021) also developed an on-chip model by co-culturing primary human hepatic cell lines (hepatocytes, KC, HSC and LSEC). In this system, lipotoxic conditions successfully generated the NASH phenotype, including hepatocellular ballooning. Furthermore, a PHH-based liver-on-a-chip modeled the progressive intracellular lipid accumulation and reduction in cytochrome P activity observed in NAFLD patient samples (Kostrzewski et al., 2020). Interestingly, the presence of the PNPLA3 I148M variant in three of the donor samples allowed the authors to highlight that this variant can worsen the NASH phenotype through effects on HSC.

Despite the numerous advantages of liver-on-a-chip platforms to study NAFLD, the liver is not the only organ involved in the disease, with adipose tissue (Stojsavljević et al., 2014) and the gut (Scarpellini et al., 2014) known to play a key role in its progression. For this reason, it is important to study the interactions between multiple organs, and their contribution to disease establishment and progression. Multi-organ systems, also known as body-on-a-chip, are emerging as technological platforms to study the efficacy and safety of drugs (Picollet-D'hahan et al., 2021; Chen et al., 2020; Skardal et al., 2020). As most of the lipids accumulated in the liver are attributed to dietary intake, Lee and Sung (2018) focused on the gut-liver axis and developed an on-chip device generating hepatic steatosis. Their gut-liver-on-a-chip device consists of two chambers, incorporating Caco-2 (gut) and HepG2 (liver) cells, separated by a porous membrane and a scaffold on the intestinal part. Using this device, Lee and Sung showed that butyrate, a compound known to enhance the gut barrier function, attenuated lipid accumulation; a benefit not observed when the drug was tested on hepatocytes alone. Recently, Slaughter et al. (2021) developed a body-on-a-chip with adipose and liver tissue, to evaluate the mechanisms involved in NAFLD development and serve as a drug screening platform. The authors were able to validate the indirect influence of adipocytes on hepatocytes in NAFLD progression. At the cost of greater complexity, the examples above hint that body-on-a-chip devices may offer a better approximation of the *in vivo* NAFLD environment.

### 3D bio-printed liver-like tissues

Three-dimensional bio-printing is another promising technique for the development of *in vitro* models. 3D structures, including liver-like tissues, are generated via layer-by-layer, spatially controlled deposition of biological materials, biochemicals and cells (van Grunsven, 2017). A comparative analysis of the three most established methods to generate 3D bio-printed liver structures (e.g., inkjet-based, extrusion-based, and photocuring-based bioprinting), is extensively reviewed elsewhere (Murphy and Atala, 2014; Ma et al., 2020). Despite notable advantages, a challenge to the development and widespread use of 3D bio-printed structures is the selection of an appropriate bio-ink. Indeed, the material must show compatibility with both cells and the printing process, while retaining physicochemical –and therefore functional– properties analogous to the organ under consideration. Alginate, gelatin, and collagen are among the most routinely used materials for this purpose.

Extensive research has been carried out on bio-printed liver structures (Norona et al., 2016; Smith et al., 2004; van Grunsven, 2017). To the best of our knowledge, however, only one 3D bio-printed system has been used to generate the NAFLD phenotype (Kizawa et al., 2017). In this study, a pathological liver tissue was bio-printed using cells harvested from genetically obese Zucker rats with NAFLD. Specifically, the Kenzan method (Moldovan et al., 2017) was used to create a scaffold-free bio-printed structure from liver spheroids composed of rat hepatocytes and mouse fibroblasts. The authors observed high lipid content over 23 days, suggesting that the pathological conditions could be retained and monitored long-term. Despite being designed to investigate drug-induced liver injury, the sophistication of the bio-printed structure proposed by Nguyen et al. (2016) makes it an ideal candidate for the study of NAFLD and NASH. This two-compartment system includes NPC – HUVEC and HSC – seeded at the boundary of each compartment, filled with PHH.

In parallel, due to the shortage of donor organs and inherent risks, researchers are exploring alternative solutions to liver transplant in patients with advanced liver diseases. Emerging approaches in development include liver-like structures bio-printed from HepG2 (Jeon et al., 2017; Ide et al., 2020) or PHH (Kim et al., 2017, 2018) that could pave the way to artificial livers in the future.

3D bio-printing technology may also enhance the assembly of liver-on-a-chip systems, which present challenges such as biological culture preparation (Bhatia and Ingber, 2014). The 3D bio-printed organ-on-a-chip of Lee and Cho (2016) was devised to overcome limitations such as spatial heterogeneity and the difficulty of providing multiple types of ECM environments when studying cell–ECM interactions (Yeon and Park, 2007). HepG2 cells encapsulated in collagen type I, and HUVEC encapsulated in gelatin were used to construct 2D and 3D hydrogel-based on-chip devices. Similarly, Bhise et al. (2016) developed a liver-on-a-chip with bio-printed HepG2/C3A spheroids. The viability of both models, their extensive life-span and similarities with intact liver tissue, makes them promising systems for both NAFLD research and drug screening pipelines.

Taken together, although use in NAFLD research is limited, these studies highlight the potential use of 3D bio-printing. However, to enable more widespread use, the costs and specialized knowledge and expertise required to establish and maintain this technology will need to be addressed (van Grunsven, 2017).

## Use of *in vitro* models in NAFLD drug development pipelines

The majority of clinical trials have focused on patients with NASH and bridging fibrosis, who have an increased risk of developing cirrhosis and adverse outcomes. Depending on the drug mechanism of action, the primary endpoint of interventional trials comprises either NASH resolution without worsening of fibrosis or a minimum of one-stage fibrosis improvement without worsening of NASH. Yet, most clinical trials have been unsuccessful and 4 out of 5 NASH treatments reaching phase III were recently terminated. This suggests monotherapies –individual drugs– might be insufficient to tackle the complex interaction between metabolic, inflammatory and fibrotic pathways that characterize this disease. As a result, evaluation of drug combinations is becoming a major focus of Pharma NASH pipelines. However, despite more than twenty years of intense research activity, no breakthrough has occurred. Indeed, the phase II ATLAS study (NCT03449446) failed to meet the primary endpoint, except for the firsocostat-cilofexor combination, currently subject of further investigation (Gilead Sciences, 2019). While a detailed discussion of the pharmaceutical compounds that have been part of clinical trials does not fall under the scope of this review, we point to extensive recent reviews that address this topic (Fallowfield et al., 2021; Campbell et al., 2021; Guirguis et al., 2021). In order to facilitate the reader's understanding of this section, we have provided two tables that summarize the most relevant information of the ongoing (Table 2) and the terminated/completed (Table 3) phase III clinical trials for pharmaceutical compounds in NAFLD.

**Table 2:** Ongoing phase III clinical trials in NAFLD and NASH

Compound/company	Study design	Mechanism of action	Intervention/control	Key inclusion criteria	Key exclusion criteria	Status
Obeticholic acid (OCA)/Intercept pharmaceuticals	REGENERATE. NASH patients (NCT02548351). Primary outcomes: ≥1 stage fibrosis improvement and no worsening of NASH or NASH resolution without worsening of fibrosis	Farnesoid X (FXR) receptor agonist	– OCA 10 mg/day (N= 330) – OCA 25 mg/day (N= 324) – Placebo (N= 312)	– NASH diagnosed by liver biopsy with 3 key histological features of NASH CRN – F2/F3 or F1 with BMI≥30/type 2 diabetes/ALT>1.5 x ULN – Stable body weight	– Model of end stage liver disease (MELD) score >12 – ALT≥10x ULN – HbA1c>9.5% – Other liver diseases – Liver transplant – Cirrhosis – BMI>45 kg/m2	Ongoing. Current results: – NASH resolution not met (p= 0.18/p= 0.13) (OCA 10 mg/OCA 25 mg) – Fibrosis improvement met (p= 0.045/p= 0.0002)
Aramchol/Galmedresearch and development, Ltd.	ARMOR. NASH (NCT04104321). Primary outcomes: ≥1 stage improvement fibrosis without worsening of NASH or NASH resolution without worsening fibrosis	Staroyl coenzyme A desaturase 1 inhibitor	–Aramchol 300 mg/day – Placebo (N total= 2000)	– NASH diagnosed by liver biopsy – NAS≥4 with at least 1 on each NAS component – F2/F3 – BMI 25–40 kg/m2 – AST>20 IU/L	– Cirrhosis – MELD score>12 – Other liver diseases – Weight loss>5% in last 3 months – Bariatric surgery – Treatment with anti-diabetic medications	Ongoing
Resmetirom (MGL-3196)/Madrigal pharmaceuticals, Inc.	MAESTRO-NAFLD1 NAFLD patients (NCT04197479). Primary outcomes: assess the effect of daily oral admin.	Thyroid hormone receptor-β agonist	–Resmetirom 80 mg/day –Resmetirom open label or double-blinded 100 mg/day – Placebo (N total= 700)	Suspected or confirmed NAFLD or NASH	– Other liver diseases – Cirrhosis – Bariatric surgery – HbA1c≥9% – GLP-1/vit. E/pioglitazone therapy – MELD>12 – ALT>250 U/L – Weight change>5%	Ongoing
Resmetirom (MGL-3196)/Madrigal pharmaceuticals, Inc.	MAESTRO-NASH. NASH patients (NCT03900429). Primary outcomes: NASH resolution with <2 points in NAS and no worsening of fibrosis	Thyroid hormone receptor-β agonist	–Resmetirom 80 mg/day –Resmetirom 100 mg/day – Placebo (N total= 2000)	Biopsy proven NASH with NAS≥4 and at least 1 on each component and F1/F2/F3	– Other liver diseases – Cirrhosis – Bariatric surgery – HbA1c≥9– GLP-1/vit. E/pioglitazone therapy – MELD>12 – ALT>250 U/L	Ongoing
Resmetirom (MGL-3196)/Madrigal pharmaceuticals, Inc.	MAESTRO-NAFLD-OLE. NAFLD patients (NCT04951219). Primary outcomes: assess the effect of daily oral admin.	Thyroid hormone receptor-β agonist	–Resmetirom 80 mg/day first 12 w followed by open-label –Resmetirom 100 mg/day –Resmetirom 100 mg/day first 12 w followed by open-label –Resmetirom 100 mg/day – Open-label Resmetirom 100 mg (N total= 1000)	– Participated in MAESTRO-NAFLD-1 – Liver biopsy with NAS = 3 and F2/F3 or NAS≥4 in all NAS components and F1, PRO-C3≤14	– Other liver diseases – HCC	Ongoing
Lanifibranor (IVA337)/Inventiva Pharma	NATiV3: NASH patients with F2/F3 (NCT04849728). Primary outcomes: resolution of NASH and improvement of fibrosis of ≥1 according to NASH CRN (Part 1). Assess the effect on delaying NASH disease progression measured by a composite endpoint that includes progression to cirrhosis, liver-related clinical outcome events, or all-cause death	Pan-PPAR agonist	–Lanifibranor 800 mg/day –Lanifibranor1200 mg/day – Placebo (N total= 2000)	– Liver biopsy with steatosis score ≥1, act. score A3/A4 and F2/F3 according to Steatosis-Activity-Fibrosis (SAF) – Stable dose of GLP-1, vit. E or statins – No weight change ≥5%	– Other chronic liver disease – HCC or cirrhosis – HbA1c>9% – Bariatric surgery	Ongoing
Semaglutide/Novo Nordisk A/S	NASH patients with F2/F3 (NCT04822181). Primary outcomes: resolution of NASH and no worsening of fibrosis or improvement of fibrosis and no worsening of NASH (Part 1). Assess the effect on delaying NASH disease progression measured by a composite endpoint that includes progression to cirrhosis, liver-related clinical outcome events, or all-cause death	Glucagon-like peptide-1 receptor agonist (GLP-1 RA)	– Semaglutide 1 subcutaneous admin./week – Placebo (N total= 1200)	– Liver biopsy with NASH – F2/F3 according to NASH CRN System – NAS score≥4 with 1 score in steatosis, inflammation and ballooning	– Other liver diseases – Treatment with pioglitazone, vit. E or other glucose-lowering agents	Ongoing

**Table 3:** Terminated and completed phase III clinical trials in NAFLD and NASH

Compound/Company or academic lead	Study design	Mechanism of action	Intervention/control	Key inclusion criteria	Key exclusion criteria	Status
Metadoxine/Hospital General de Mexico	Non-diabetic patients with NAS>3 (NCT02541045). Primary outcome: Improvement of NAS	–↓Oxidative stress – Restoration NADH, ATP and GSH – Prevention increase collagen –↓TNF secretion	–Metaxodine 500 mg/day – Placebo (N total= 108)	– Non-diabetic patients – BMI≥25 – Proof of liver steatosis with ultrasonography – NAS≥3 with at least 1 point on each – Without fibrosis or fibrosis stage ≤ F2 according to NASH CRN classification	– Cirrhosis – Other liver diseases – Uncontrolled chronic disease, hypothyroidism or hyperthyroidism	Suspended due to lack of financial resources
Rimonabant (SR141716)/Sanofi	NASH patients with T2DM (NCT00577148) and without (NCT00576667)	Cannabinoid-1 receptor blocker	– Rimonabant 20 mg/day – Placebo (N total= 89 for NCT00577148and N= 165 for NCT00576667)	NASH patients	– T1DM for NCT00577148 or type I/II diabetes for NCT00576667 – Other chronic liver disease – Previous or current HCC – Previous bariatric surgery	Terminated by company decision taken in light of demands by certain national health authorities
Cenicriviroc (CVC)/AbbVie	AURORA: NASH patients with liver fibrosis (NCT03028740) Primary outcomes: Improvement of ≥1 stage in fibrosis according to NASH CRN System without worsening of NASH (Part 1); and improvement of histopathological progression, liver-related clinical outcomes and all-cause mortality (Part 2)	Chemokine 2 and 5 receptor antagonist	– CVC 150 mg/day – Placebo (N total= 1779)	– Proof NASH based on liver biopsy – Subjects in part 1: F2/F3 per the NASH CRN System based on liver biopsy. Subjects in Part 2: F3 per the NASH CRN System, based on liver biopsy.	– Other liver diseases or serious infections – Liver transplantation – HbA1c>10% at Screening – Weight reduction ≥7% through bariatric surgery in the past 5 years or bariatric surgery planned during study – Malignancy within past 5 years or ongoing, other than basal cell carcinoma or resected noninvasive cutaneous squamous cell carcinoma – GLP-1 agonist, DPP-4 inhibitor, SGLT2 and/or SGLT1 inhibitor, TZD for ¡6 mo. before screening	Terminated due to lack of efficacy in Part I
Elafibranor (GTF505)/Genfit	RESOLVE-IT: NASH patients with fibrosis (NCT02704403). Primary outcomes: NASH resolution without worsening of fibrosis; composite long-term outcome composed of all-cause mortality, cirrhosis, liver-related clinical outcome	Peroxisome proliferator-activated receptor-α and peroxisome proliferator-activated receptor-δ agonist	– Elafibranor 120 mg/day – Placebo (N total= 2157)	– BMI≤45 kg/m^2^ – NASH confirmation by liver biopsy with at least 1 in each component NAS score – NAS score≥4 – Fibrosis stage≥1 or <4 according to the NASH CRN system	– Known heart failure – Bariatric surgery – Uncontrolled hypertension – T1DM patients – HbA1c>9% – Weight loss >5% within last 6 months – Compensated and decompensated cirrhosis – Other chronic liver diseases	Terminated due to failure to meet primary efficacy endpoint
Selonsertib (SEL/GS-4997)/Gilead Sciences	NASH patients and F3 (NCT03053050) Primary outcomes: ≥1 stage improvement in fibrosis according to NASH CRN System and event-free survival	ASK-1 inhibitor	– SEL 6 mg/day – SEL 18 mg/day – Placebo (N total= 808)	– Liver biopsy NASH and F3 according to NASH CRN System – HbA1c≥9.5– ALT ≤ x8 ULN	– MELD score >12 – Other liver diseases – Liver transplantation – HCC	Terminated due to lack of efficacy
Diamel/Catalysis SL	NASH with insulin resistance (NCT00820651). Primary outcome: histological improvement	– Antioxidant – Biocatalyst –↓ Gastrointestinal absorption glucose	–Diamel 660 mg/x2 every 8h – Placebo: hypocaloric diet of 1620 kcal daily (N total= 158)	Histological diagnosis of NASH	– Other liver disease – Decompensated cirrhosis – Fasting glucose levels >250 mg/dL	Completed (no results available)
Pioglitazone or vit. E/National Institute of diabetes and Digestive and Kidney diseases (NIDDK)	PIVENS: NASH (NCT00063622). Primary outcomes: improvement of ≥1 hepatocyte ballooning, NAS≤3 or decrease of 2 points and no worsening of fibrosis	– Vit. E: antioxidant – Pioglitazone: thiazolidinedione that targets insulin resistance and adipose tissue dysfunction	– Pioglitazone 30 mg/day (N= 80) – Vit. E 800 IU/day (N= 84) – Placebo (N= 83)	NASH based on liver biopsy	– Adults with diabetes – Other liver or cardiovascular diseases – Cirrhosis	Completed. Significant difference for vit. E (p= 0.001), but not with pioglitazone (p= 0.04). Improvement in serum ALT and AST (p <0.001), steatosis (p= 0.005/p= 0.001) (vit. E/pioglitazone) and inflammation (p= 0.02/p= 0.004); but not fibrosis scores (p= 0.24/p= 0.12). (Pre-specified level of significance was p= 0.025)
Losartan/Newcastle-upon-Tyme Hospitals NHS Trust	FELINE: NASH patients (NCT01051219). Primary outcome: change in histological fibrosis stage (NASH CRN system)	Angiotensin II receptor type 1 (AT1) antagonist	– Losartan 50 mg/day (N= 15) – Placebo (N= 17)	NASH and fibrosis stage 1–3 NASH CRN System	– Use of ACEI or ARBs in past year – Change in diabetes regimen in last 3 months – Weight loss >50% in last 6 months	Study was terminated early due to slow recruitment, but patients were allowed to complete the study if they wanted to. No significant results
Oltipraz/PharmaKing	NAFLD patients (NCT02068339). Primary outcomes: change in liver fat assessed by MRS	AMP-activated protein kinase (AMPK) activator	–Oltipraz 90 mg/day –Oltipraz 120 mg/day – Placebo (N total= 283)	– NAFLD patients – Abnormal ALT/AST	– Cirrhosis – AST/ALT >2 ratio – T1DM/T2DM – Other liver diseases/cancer – Vit. E consumption – Bariatric surgery within 6 months	Completed. No results available
Pentoxifylline (PO TID)/NortwesternUniversity	NASH patients (NCT00267670). Primary outcomes: Improvement of ALT≥30% change	Non-specific phosphodiesterase inhibitor	– PO TID 400 mg/day (N= 19) – Placebo (N= 7)	– Steatosis score ≥1 – ALT ≥ x1.5 ULN – HgbA1c<7%	– Decompensated cirrhosis – Current anti-diabetic, anti-TNF-α or vit. E medication	Completed. No significant change in ALT (p= 0.08)

*In vitro* cell culture assays have been used to support pre-clinical development of candidate therapies and to validate the utility of new cellular models. For instance, obeticholic acid (OCA) has been tested in human iPSC-derived hepatocytes (Parafati et al., 2018), a co-culture of human hepatoma cells (Huh7) and HSC (LX-2) (Anfuso et al., 2020) and an organotypic human hepatocyte system (Dash et al., 2017). Boeckmans et al. (2020) investigated the properties of several PPAR agonists in PHH, HepaRG, HepG2, LX-2 and human skin stem cell-derived hepatic cells. Other studies, include the use of human HSC (HSC-T6 and LX-2) to determine the efficacy of selonsertib in liver fibrosis (Yoon et al., 2020) or primary rat HSC to evaluate the effects of pioglitazone *in vitro* (Kawaguchi et al., 2004).

Additionally, fibrogenic spheroids have been used to test the efficacy of putative drugs (e.g., sorafenib (Romualdo et al., 2021), lanifibranor, elafibranor, or cenicriviroc (Hurrell et al., 2020)) in mediating resolution of NASH, thereby validating the role of spheroids for drug screening.

Beyond being suitable models to elucidate NAFLD pathogenesis, liver-on-a-chip systems also play a key role in drug development. To date, Gori et al. (2016), Freag et al. (2021), Cho et al. (2021), Du et al. (2021) and Gori et al. (2021) have used liver-on-a-chip devices to test the ability of polyphenols, OCA, elafibranor, and pirfenidone to ameliorate NAFLD. Interestingly, while the disease-modifying effects of elafibranor were shown in a liver-on-a-chip assay, this did not translate to clinical efficacy in a pivotal phase III NASH trial. As a consequence, the drug program was recently terminated (GENFIT, 2020). These contrasting *in vitro* versus *in vivo* observations indicate that preclinical NAFLD models still have a long way to go before reliably simulating human NAFLD and predicting patient responses to therapies.

## Translation of *in vitro* platforms

The challenge of identifying an efficacious treatment for the disease is evidenced by the litany of recent clinical trial failures that have encompassed a diverse range of targets. Notwithstanding, many synthetic drugs are currently in development as monotherapy or combination therapy regimens. The availability of reliable human-based *in vitro* models would help to address this challenge, by both enhancing our understanding of NAFLD progression and predicting the therapeutic response to drug candidates. So far, *in vitro* models have only managed to recapitulate specific aspects of the *in vivo* disease phenotype. Indeed, the establishment of multi-dimensional systems that reflect the combined effects of genetics, sex, comorbidities and inter-organ cross-talk remains a pipe dream. Mounting evidence suggests, however, that the complexity of *in vitro* models will be key to screen drugs and to predict clinically relevant therapeutic responses. This is illustrated in Lee and Sung (2018), where the beneficial effect of butyrate on lipid accumulation only became apparent in a gut-liver-on-chip, but was not detected when the compound was tested using hepatocytes alone.

An ideal NAFLD *in vitro* model used for drug screening should satisfy biological fidelity and usability requirements. Biological fidelity depends on the selection of appropriate liver cells (parenchymal cells and NPC) and their aggregation in a structure enabling cell-cell and cell-ECM interactions. Future trends will likely see a transition from immortalized cell lines to PSC, due to their almost unlimited supply, life-span and similarity to *in vivo* human phenotype (Hay et al., 2007; Gao and Liu, 2017). For the observed response to be shaped by inter-organ cross-talk, these systems need to be linked with intestinal, pancreatic and adipose tissue models. 3D liver structures cultured in perfusion devices are quickly developing to meet these standards. Usability of such models relies instead on their lifetime (e.g., a minimum of 2–3 weeks of observation are needed for cytotoxicity testing) and the availability of specialized know-how for their operation.

To ensure clinical relevance, NAFLD *in vitro* models require validation against known features of *in vivo* human pathogenic processes or responses to therapeutic agents. Such validation could be performed via an ‘omics’ approach, which can evaluate the degree of pathological equivalence between an *in vitro* model and human liver samples at the molecular level, as measured by differentially expressed genes and enriched pathways. Such a strategy has been adopted by Ramli et al. (2020) and Feaver et al. (2016).

**Figure 4:**
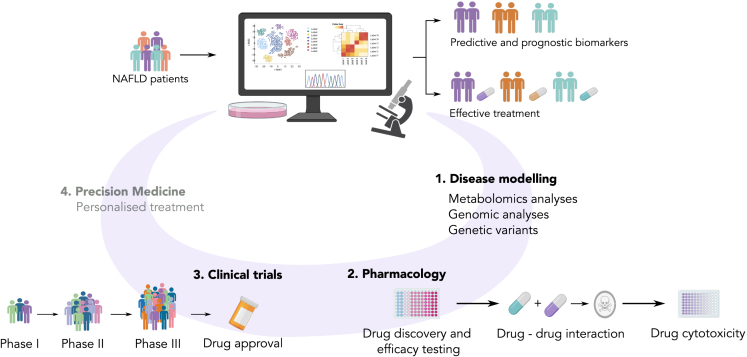
Potential applications of *in vitro* models in translational research for NAFLD The combination of ‘omics’ and cell cultures has the potential to enable the study NAFLD patients ‘in-the-dish’, resulting in the design of effective treatment or identification of predictive and prognostic biomarkers. *In vitro* NAFLD models, combined with metabolomic and genomic analyses/genetic variant studies, permit the identification of pharmacological targets and susceptible patient subpopulations. Once a putative drug – or combination of drugs – has been indicated as a candidate NAFLD therapy, several pharmacological investigations test the efficacy, cytotoxicity, and possible interactions between compounds. Drugs that are successfully triaged are funneled to clinical evaluation. The ultimate goal –which has not yet been achieved– would employ a precision medicine approach (gray) to the design of patient-tailored NAFLD treatments.

Alternatively, models could be assessed by their ability to reproduce results obtained in ongoing clinical trials. For example, Hurrell et al. (2020) demonstrated the potential of their system as a drug screening platform by testing pharmaceuticals that were in phase II or phase III clinical development. However, robust human validation and critical comparative analyses are rarely performed, with most researchers focusing on specific genes and pathways of interest (e.g., the expression level of TGF-α, α-SMA or TNF-β eliciting fibrosis and inflammation) when designing *in vitro* systems for NAFLD. Ideally, extensively validated platforms would allow more reliable pharmacodynamic-pharmacokinetic and toxicity assays of candidate compounds for NAFLD treatment, de-risking subsequent clinical trials. Several drugs that are currently in phase III have undergone preliminary *in vitro* testing. For instance, Aramchol was tested in bioassays on PHH, HSC, and pHSC (Fernández-Ramos et al., 2020; Bhattacharya et al., 2021). While not using 3D *in vitro* models, the authors selected multiple cell lines to investigate genes and pathways modulated by Aramchol (e.g., downregulation of SCD1, COL1A1, and ACTA2; upregulation of PPARG). This cost-effective strategy benchmarks a putative drug according to multiple aspects of NAFLD, before proceeding to test its efficacy in suitable cohorts of patients. Similarly, *in vitro* studies with OCA in co-cultures of Huh7 and HSC (Anfuso et al., 2020) or PHH collagen gel sandwich (Dash et al., 2017), have delineated mechanisms of action including modulation of collagen and MMP2 and 9 activity, as well as its possible toxicological effects. Figure 4 shows the different potential applications of *in vitro* models in translational research for NAFLD.

Despite the advantages they offer, 3D *in vitro* models remain expensive and inaccessible to new users. Indeed, liver-on-a-chip and bio-printed liver-like tissues are quite complex and steep learning curves are associated with their operation. Ideally, technological advancements and the establishment of rigorous standards will enable these models to become a concrete alternative to animal models in the near future.

## Future perspectives

Emerging *in vitro* human-based models have enhanced our understanding of NAFLD evolution and disease mechanisms. However, open challenges remain. Among these, the most prominent are the ability of the model to encompass the whole spectrum of the disease, to reproduce the inter-individual variability observed in NAFLD patients and capture the interaction between multiple organs involved in the pathogenesis of the disease. We expect that in the near future complex *in vitro* models, such as body-on-a-chip based on iPSC or PHH, will become the platform of choice to mimic NAFLD establishment and progression. Inclusion of physiologically-based toxicokinetic computer modeling to study ADME (absorption, distribution, metabolism, and excretion) in these systems will likely represent a further step toward the replication of the human liver microenvironment.

The availability of validated preclinical models will streamline the adoption of a quantitative approach to (a) the identification of NAFLD etiology and potential therapeutic targets, and (b) the rational design of pharmacological treatments. These aims are increasingly pursued in a biological systems control perspective, wherein a disease arises due to failure of multi-scale control mechanisms ensuring robustness of biological processes to internal/environmental perturbations. Using this approach, mathematical models and gene regulatory networks reconstructed from ‘omics’ data provide new insights on the establishment of diseases; and the design of personalized effective treatments can be framed as an engineering control problem. For example, Liao et al. (2020) used a kinetic model of hepatic fructose metabolism to simulate the effect of excessive dietary uptake of this sugar on dyslipidemia observed in early NAFLD patients. The authors identified fructokinase as a molecular target of the fructose pathway and predicted its suppression would revert lipid accumulation. Van Riel et al. (2020) used instead a machine learning algorithm, informed by metabolomic and transcriptomic time-series, to predict the metabolic response induced by treatment with a Liver X Receptor (LXR) agonist. Currently, 3D models are mainly used for biological studies due to their cost and low-throughput. Tackling such limitations will prompt adoption of these *in vitro* models by the pharmaceutical industry. Indeed, within drug development pipelines, these models would allow only the most promising compounds to funnel toward clinical trials, with obvious economic benefit. If used in combination with disease-relevant *ex vivo* models (e.g., human precision-cut liver slices) (Palma et al., 2019), *in vitro* models might help to reduce the (over)reliance on *in vivo* models for drug efficacy and toxicological studies.

We expect complex *in vitro* platforms to be increasingly integrated with ‘big data’ for compound testing, identification of therapeutic targets, and personalized medicine applications. Precision medicine approaches would enable, first, the clustering of NAFLD patients by disease stage and/or genetic variants (e.g., PNPLA3) and the development of *in vitro* models from patient-derived iPSC on which to assess safety and efficacy of candidate therapies. Second, these studies could identify novel companion biomarkers of NAFLD progression or regression, thereby supporting the design of non-invasive diagnostic tests.

In conclusion, we have provided a comprehensive overview of various *in vitro* models proposed to study the pathophysiology of NAFLD. The different models available for this disease have specific advantages and disadvantages. Accordingly, the choice of model may depend on the particular context of use.

## Supplemental information

Document S1. Figures S1 and S2 and Table S1

Table S1. List of final articles used for the systematic review This table includes the title of the publication, the year it was published, and the journal where it was published.
