# Supplementary material for: In vitro models for non-alcoholic fatty liver disease: Emerging platforms and their applications
Source: iScience. 2021 Dec 4;25(1):103549. doi: 10.1016/j.isci.2021.103549 (PMC8689151; doi:10.1016/j.isci.2021.103549)
Supplement: Document S1. Figures S1 and S2 and Table S1 [file mmc1.pdf]

iScience, Volume *25*

Supplemental information

In vitro models for non-alcoholic fatty liver disease: Emerging platforms and their applications

Maria Jimenez Ramos, Lucia Bandiera, Filippo Menolascina, and Jonathan Andrew Fallowfield

1. Figures

Figure S1. Flow diagram of the systematic analysis.

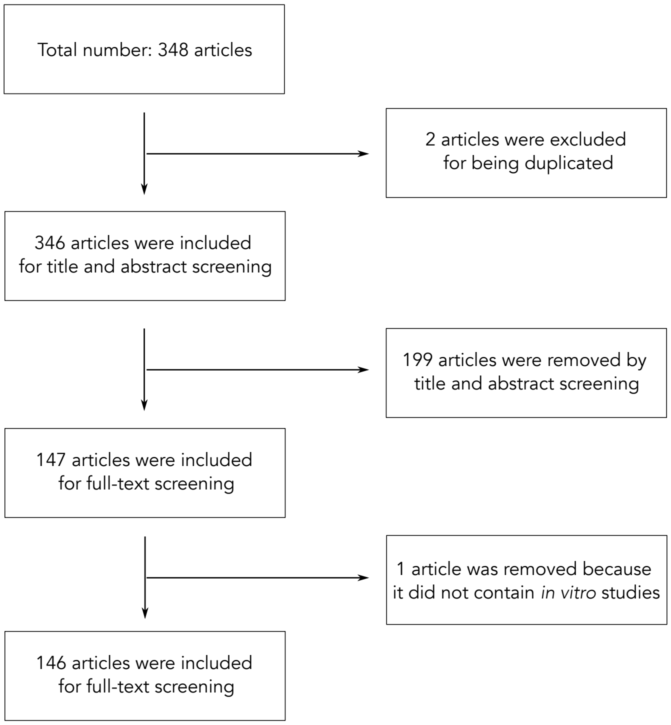

Figure S1: Flow diagram of the search strategy of publications focussing on *in vitro* NAFLD models.

Figure S2. Evolution over time of the fraction of publications focusing on each *in vitro* model.

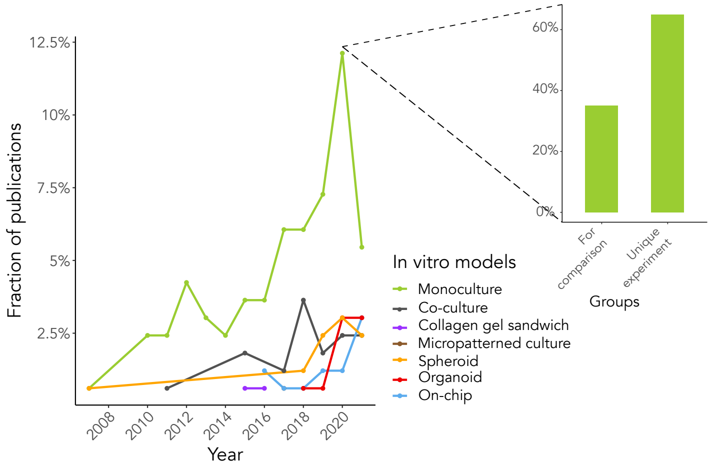

Figure S2: Monocultures (green), co-cultures (black), collagen gel sandwich (purple), micropatterned cultures (brown), spheroids (orange), organoids (red), on-chip (blue). Whilst 2D culture is the predominant method, the past four years saw an increased availability of 3D NAFLD *in vitro* models. In the inset, a barplot quantifies the number of studies, published in 2020, that have used monocultures as the primary *in vitro* model, or as a standard reference to validate more complex alternatives.

2. Tables

Table S1. List of final articles used for the systematic review. This table includes the title of the publication, the year it was published and the journal where it was published.
